# Plasma soluble PD-L1 and STAT3 predict the prognosis in diffuse large B cell lymphoma patients

**DOI:** 10.7150/jca.47816

**Published:** 2020-10-17

**Authors:** Yue Fei, Jingwei Yu, Yang Li, Linyu Li, Shiyong Zhou, Tingting Zhang, Lanfang Li, Lihua Qiu, Bin Meng, Yi Pan, Xiubao Ren, Zhengzi Qian, Xianhuo Wang, Huilai Zhang

**Affiliations:** 1 Department of Lymphoma, Tianjin Medical University Cancer Institute and Hospital, National Clinical Research Center of Cancer, Key Laboratory of Cancer Prevention and Therapy, Tianjin's Clinical Research Center for Cancer, the Sino-US Center for Lymphoma and Leukemia Research, Tianjin, China.; 2 Department of Radiotherapy, The Second People Hospital of Dezhou, Shandong, China.; 3 Department of Pathology, Tianjin Medical University Cancer Institute and Hospital, Tianjin, China.; 4 Department of Immunology/Biotherapy, Tianjin Medical University Cancer Institute and Hospital, Tianjin, China.

**Keywords:** sPD-L1, STAT3, DLBCL, prognosis, biomarker

## Abstract

**Purpose:** Diffuse large B cell lymphoma (DLBCL) is one of the most common B cell lymphomas, which displays heterogeneous pathologies. Programmed cell death 1/ programmed cell death ligand 1 (PD-1/PD-L1) plays an essential role in immunosuppression in multiple malignancies. Signal transducer and activator of transcription 3 (STAT3)-positive patients also have an independently inferior clinical outcome. However, there are no reports on the effect of plasma soluble PD-L1 (sPD-L1) combined with plasma STAT3 on the prognosis of DLBCL. In this study, we investigate the relationships between plasma sPD-L1 combined with STAT3 and clinical prognosis of DLBCL.

**Methods:** Levels of plasma sPD-L1 and STAT3 were quantified using ELISA in eighty-seven DLBCL patients. Multiplexed immunofluorescence staining was performed to visualize the expression of PD-L1 in twenty-nine matched FFPE specimens from all patients.

**Results:** The survival analysis revealed that the progression-free survival (PFS) and overall survival (OS) in high sPD-L1 level group were poorer than that in low sPD-L1 level group (PFS, *P* < 0.001; OS, *P* < 0.001). Similarly, the PFS and OS in high STAT3 level group were also poorer than that in low STAT3 level group. Multivariate cox regression analysis showed that both high sPD-L1 and high STAT3 levels were the independent prognostic factors negatively affecting survival. In addition, patients with DLBCL having high levels of both sPD-L1 and STAT3 had a worse outcome than those patients having any one high or low levels of both (*P* < 0.001).

**Conclusions:** We therefore revealed that high levels of plasma sPD-L1 and STAT3 are associated with inferior outcome for DLBCL patients, suggesting that combined measurement of their levels in plasma may be a promising prognostic strategy for DLBCL patients.

## Introduction

Diffuse large B cell lymphoma (DLBCL) is one of the most common B-cell lymphomas, accounting for approximately 30-40% of non-Hodgkin's lymphoma (NHL) 1. It is characterized by a group of patients that have significant heterogeneity in clinical manifestations, pathological phenotype, and prognosis survival rate 2. In recent years, studies on various tumor immune checkpoints including PD-1/PD-L1 (programmed cell death 1/programmed cell death ligand 1) and regulatory mechanisms have been increasing immensely. Furthermore, the prognosis of several different cancer patients including those with lymphoma has been greatly improved in the era of immune checkpoint inhibitors 3-7.

PD-L1, also known as B7-H1/CD274, is an immune inhibitory receptor expressed on the surface of T cells, B cells, and monocytes upon activation. It is a member of the B7 family and an important ligand of PD-1 (also known as CD279) 8. The PD-1/PD-L1 pathway plays an important role in immune evasion by tumor cells through T-cell exhaustion. Recently, PD-L1 was observed to be overexpressed in multiple tumors including lymphoma, lung cancer, malignant pleural mesothelioma (MPM), and renal cell carcinoma 9-13. In addition, PD-L1 overexpression is associated with poor prognosis 14, 15. A previous report has shown that soluble PD-L1 (sPD-L1) is highly expressed in B cell malignant lymphoma patients compared with healthy people, especially in diffuse large B cell, small lymphocyte, mucosa-associated lymphoid tissue, and mantle cell lymphomas. However, sPD-L1 level in follicular lymphoma was low 16. Importantly, elevated sPD-L1 was associated with poor prognosis 17-18. Moreover, several studies have reported that PD-L1 expression in tissue positively correlates with plasma sPD-L1 levels in nasal natural killer/T-cell lymphoma 19 and peripheral T-cell lymphoma patients 20.

Three oncogenic pathways have been observed in DLBCL including the constitutively activated NF-κB, JAK/signal transducer and activator of transcription (STAT), and PI3K/AKT/mTOR pathways 21-23, which usually promote cell proliferation, growth, survival, and angiogenesis, and diminish apoptosis. Many factors can drive the expression of PD-L1 including STAT3 24-25. Reports displayed that nucleophosmin-anaplastic lymphoma kinase (NPM-ALK) induces PD-L1 expression by activating STAT3 26. The abnormal activation of the phosphates and tensin homolog (PTEN) gene also results in the JAK/STAT3 signaling pathway activation, further leading to upregulation of PD-L1 expression. PD-L1 expression further acts to drive tumor cell proliferation, survival, invasiveness, and metastasis 27. Patients with STAT3 overexpression were found to have significantly poorer progression-free survival (PFS) and overall survival (OS) 28-33, and some studies showed that STAT3-positive patients had an independently inferior clinical outcome 34.

Although high level of plasma sPD-L1 is associated with poor prognosis in DLBCL patients 17, there are no reports on the effect of plasma sPD-L1 combined with plasma STAT3 on the prognosis in DLBCL patients. The present study aimed to investigate the relationships between plasma sPD-L1 combined with plasma STAT3 levels and clinical prognosis in DLBCL patients. These findings may provide an insight into convenient prognostic biomarkers for and risk stratification in DLBCL patients.

## Materials and Methods

### Study participants and sample collection

Our study included eighty-seven patients with de novo DLBCL whose peripheral blood was available and received R-CHOP (rituximab, cyclophosphamide, doxorubicin, vincristine, and prednisone) or R-CHOP-like regimen. Plasma samples from the peripheral blood of all patients were collected before systematic treatment and frozen at -80 °C until further analyses. Meanwhile, 29 matched formalin-fixed paraffin-embedded (FFPE) tissue from patients before treatment were collected. This study was approved by the Clinical Research Ethics Board of Tianjin Medical University Cancer Institute and Hospital (Tianjin, China). All patients provided written informed consent, and this study was conducted in accordance with the principles of Declaration of Helsinki and Good Clinical Practice guidelines.

### Quantification of plasma sPD-L1 and STAT3

Levels of plasma sPD‐L1 and plasma total STAT3 were measured using enzyme linked immunosorbent assay (ELISA) kits namely, Human PD-L1 ELISA kit (DB7H10, R&D Systems, Minneapolis, MN, USA) and Human Signal Transducer and Activator of Transcription 3 (STAT3) ELISA Kit (ml728930, Mlbio, Shanghai, China) according to the manufacturer's instructions. Each sample was analyzed in duplicates. The intra-assay and inter-assay coefficients of variation were below 20%.

### Multiplexed immunofluorescence staining for PD-L1 expression

In order to precisely detect the PD-L1 and PAX-5 (paired box 5) expression, multiplexed immunofluorescence staining was performed in twenty-nine matched formalin-fixed paraffin-embedded (FFPE) tissues from patients before treatment according to the Opal immunostaining protocol, as described in our previous study 35. Each FFPE tumor section needed two sequential rounds of staining. The primary antibodies used in this study included clone E1L3N for PD-L1 (1/ (200×5), Rabbit, Cell Signaling Technology, Danvers, MA, USA) and clone sp34 for PAX-5 (1/ (400×5), Rabbit, Ventana Roche, Oro Valley, AZ, USA). The stained signal was amplified using Opal 520 tyramide signal amplification (TSA) reagents (PerkinElmer, Waltham, MA, USA) for anti-PAX-5 and Opal 570 for anti-PD-L1. After two rounds of staining, the sections were counterstained with DAPI (Life Technologies, Carlsbad, CA, USA), mounted on Vectashield hardset medium (Life Technologies, Carlsbad, CA, USA), and stored in a light protection box at 4 °C prior to imaging. Multiplex-stained sections were imaged using the Mantra System (PerkinElmer) and the color-based identification of all markers of interest was facilitated by a single image using inform 2.3 image analysis software (PerkinElmer). DLBCL tumor cells were identified as PAX-5^+^. The mean value of PD-L1 positivity across images acquired from 20 fields of view was calculated. Phenotyping and quantification were carried out by a researcher who was blinded to the sample identities and clinical outcomes.

### Statistical analyses

We used SPSS 25.0 statistical software (IBM SPSS Statistics, Chicago, IL, USA) and Graphpad Prism 8.0 (GraphPad Software, San Diego, CA, USA) to perform all statistical analyses. Pearson's chi-squared test was used to analyze the correlations between plasma sPD-L1 and plasma STAT3 levels. The relationships between clinicopathologic parameters and sPD-L1 or STAT3 levels were analyzed via chi-square test. The association between plasma sPD-L1 and PD-L1 expression in tumor tissue was assessed by linear regression analysis. Progression-free survival (PFS) was calculated from the date of diagnosis to that of disease progression, relapse, or death from any cause. Overall survival (OS) was calculated from the date of diagnosis to that of last follow-up or death. PFS and OS were estimated using the Kaplan-Meier method and log-rank tests were performed. *P* < 0.05 was considered statistically significant.

## Results

### Patient characteristics

Table 1 summarizes the baseline characteristics of eighty-seven patients with DLBCL. The median age was fifty-six years (range: 21-83 years). Of the eighty-seven patients, forty-one (47.1%) were male, forty-two (48.3%) had the advanced disease stage, twenty-seven (31%) were middle-high or high risk, fifty-six (64.3%) with extra nodal involvement, thirty-eight (43.7%) had elevated lactate dehydrogenase (LDH) levels, and seventeen (19.5%) had elevated beta 2 microglobulin (β2-M) levels. According to the 2016 WHO classification, thirty-nine (44.8%) were diagnosed as germinal center B-cell (GCB)-DLBCL and forty-eight (55.2%) as non-GCB-DLBCL. Using cutoff values of MYC (40%) and BCL2 (50%) positive tumor cells, twenty-eight (32.2%) were diagnosed as double-expressor lymphoma (DEL), thirty-two (36.8%) as non-DEL and twenty-seven (31%) as unknown. No double hit lymphoma (DHL) or triple hit lymphoma (THL) patients were found. According to the 2014 Lugano criteria, fifty (57.5%) attained complete response (CR)/partial response (PR) and thirty-seven (42.5%) appeared with stable disease (SD)/progressed disease (PD).

### Correlation between plasma sPD-L1, STAT3 level and clinical characteristics

Using a cut-off value of median sPD-L1 level, 1.21 ng/ml, we separated the patients into two groups, which is similar to the previous study 17. Forty-four patients (50.6%) were categorized as the high sPD-L1 level group (≥ 1.21 ng/ml), and the remaining forty-three cases (49.4%) as low sPD-L1 level group (< 1.21 ng/ml). Similarly, we categorized forty-four patients as the high STAT3 level group (≥ 541.87 pg/ml), and the rest as low STAT3 level group (< 541.87 pg/ml). Table 1 showing the relationships between sPD-L1, STAT3 levels and patient clinical characteristics reveals that patients having higher sPD-L1 level were always accompanied by the advanced stage (*P* = 0.013), elevated LDH (*P* = 0.012) and β2-M levels (*P* = 0.001), international prognostic index (IPI) score > 2 (*P* = 0.003), non-GCB subtype (*P* = 0.042), B symptom (*P* = 0.012), DEL (*P* = 0.021), and poor clinical response (*P* = 0.002). High STAT3 levels were related to the advanced stage (*P* = 0.041) and IPI score > 2 (*P* = 0.044). Furthermore, we also found that there were no correlations between plasma sPD-L1 level and plasma STAT3 level (R = 0.195, *P* = 0.071).

### Correlation between plasma sPD-L1, STAT3 level and survival

**Figure 1:**
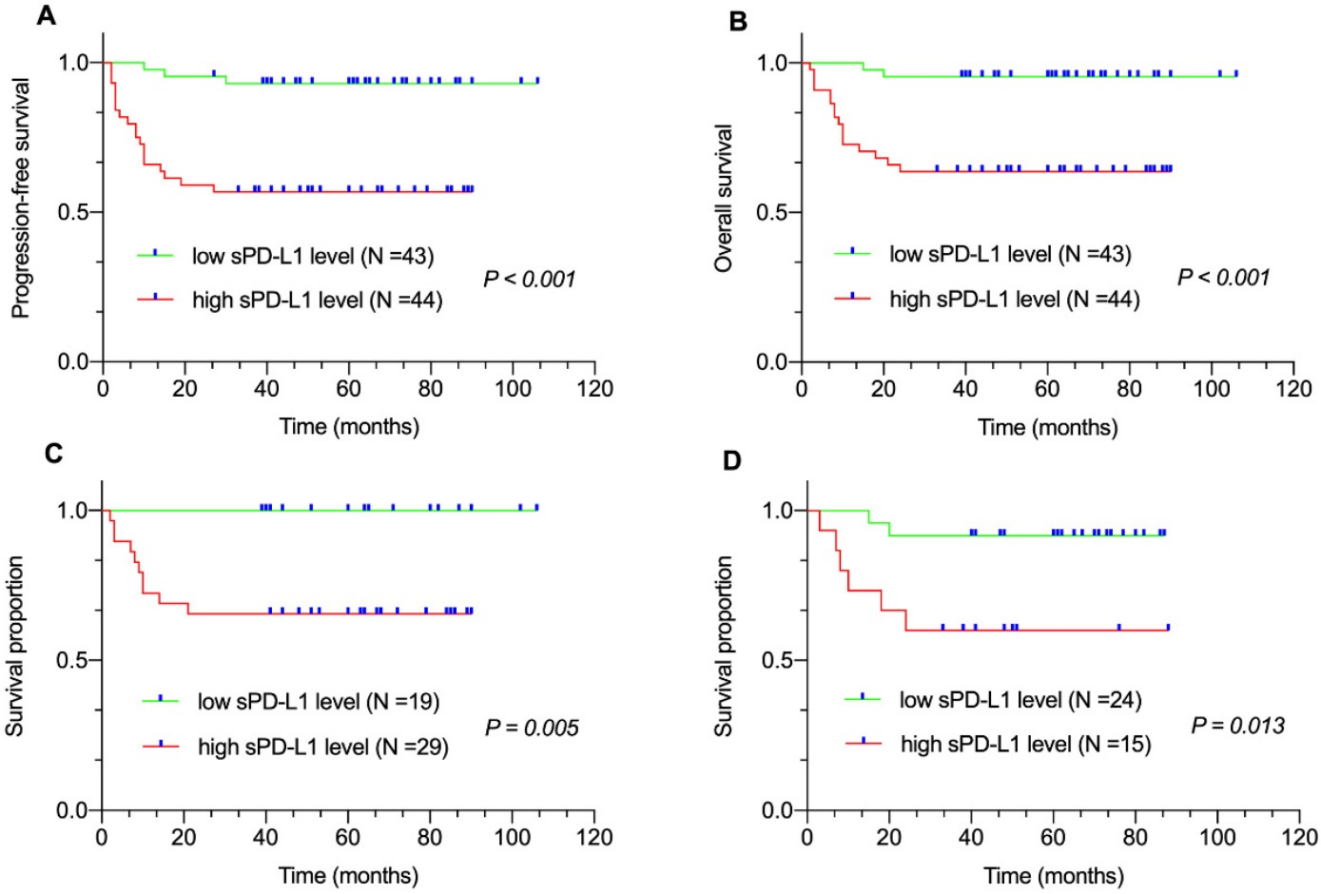
Correlation between plasma sPD-L1 level and survival. (A) Progression-free survival (PFS) and (B) Overall survival (OS) of patients with DLBCL by plasma sPD-L1 levels. Survival proportion of patients with (C) non-GCB DLBCL and (D) GCB-DLBCL by plasma sPD-L1 levels.

**Figure 2:**
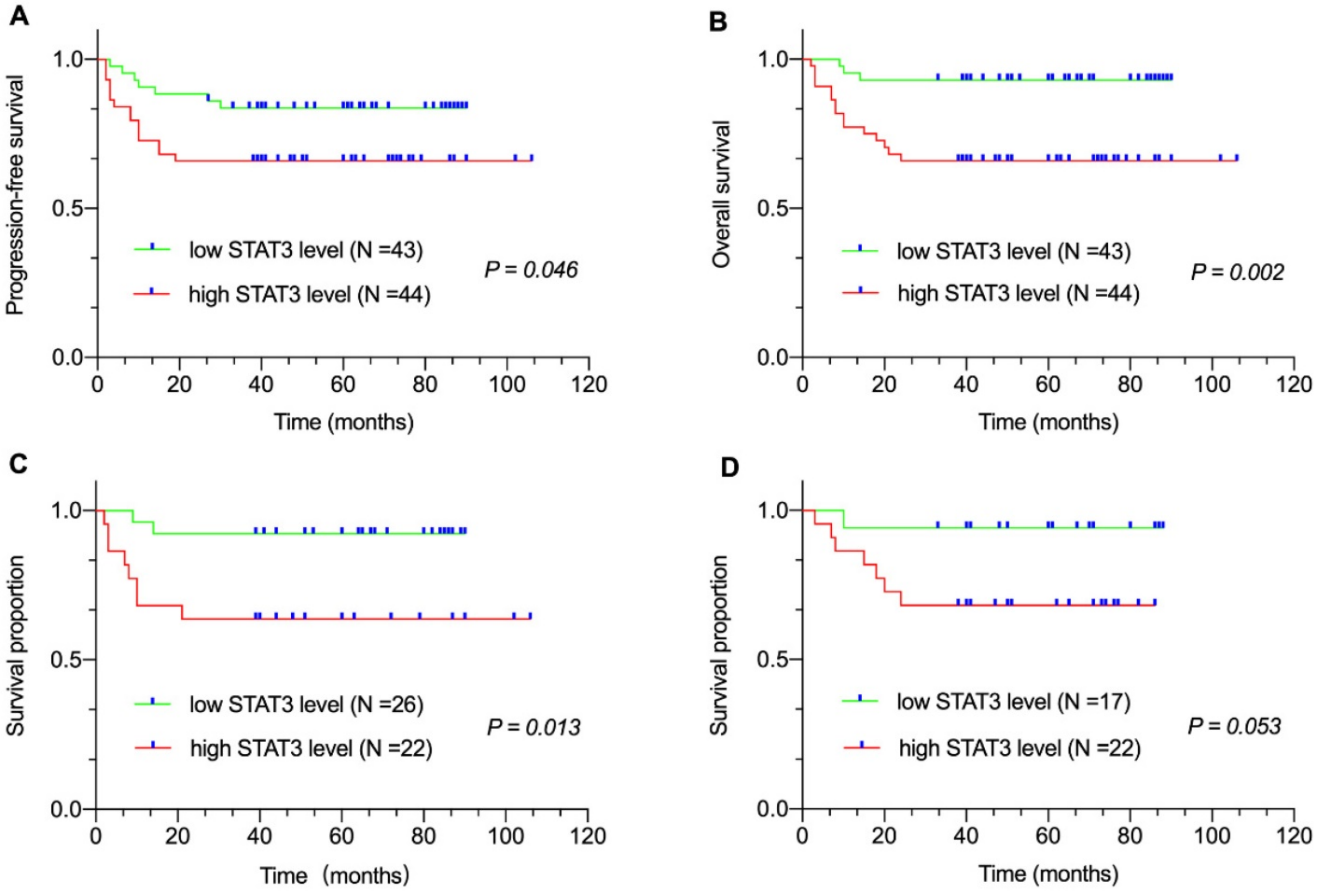
Correlation between plasma STAT3 level and survival. (A) Progression-free survival (PFS) and (B) Overall survival (OS) of patients with DLBCL by plasma STAT3 levels. Survival proportion of patients with (C) non-GCB DLBCL and (D) GCB-DLBCL by plasma STAT3 levels.

Median follow-up duration was sixty (range: 2-106 months) months. All eighty-seven patients were available for the 3-year PFS and OS analysis. We found that 3-year PFS and 3-year OS rates were 74.7% and 79.3%, respectively. The survival analysis revealed that patients with high sPD-L1 level in the entire cohort (PFS, *P* < 0.001, Figure 1A; OS, *P* < 0.001, Figure 1B) as well as in the non-GCB-DLBCL (*P* = 0.005, Figure 1C) and GCB-DLBCL subgroups (*P* = 0.013, Figure 1D) had inferior survival compared with that of patients with low sPD-L1 level. Meanwhile, patients with high STAT3 level in the entire cohort (PFS, *P* = 0.046, Figure 2A; OS, *P* = 0.002, Figure 2B) as well as in the non-GCB-DLBCL subgroup (*P* = 0.013, Figure 2C), but not in the GCB-DLBCL subgroup (*P* = 0.053, Figure 2D), had inferior survival compared with that of patients with low STAT3 level.

Subgroup analyses were performed according to gender, age, clinical stages, LDH level, β2-M level, IPI grades, B symptoms, sPD-L1 level, and STAT3 level. Table 2 summarizes the univariate and multivariate cox analyses. In the univariate analysis, LDH (*χ^2^* = 5.515; *P* = 0.019), β2-M (*χ^2^* = 11.232; *P* = 0.001), IPI scores (*χ^2^* = 15.672; *P* = 0.000), B symptom (*χ^2^*= 8.660; *P* = 0.003), DEL (*χ^2^* = 4.439; *P* = 0.035), high sPD-L1 level (*χ^2^* = 13.708; *P* = 0.000), high STAT3 level (*χ^2^* = 9.780; *P* = 0.002) were associated with poor prognosis in patients with DLBCL. Multivariate cox regression models were further performed to determine the prognostic value of sPD-L1 and STAT3 level. We found that IPI score [HR (95% CI) = 3.121 (1.124-8.669), *P* = 0.029], high sPD-L1 level [HR (95% CI) = 6.284 (1.390-28.397), *P* = 0.017], and high STAT3 level [HR (95% CI) = 4.158 (1.182-14.627), *P* = 0.026] were the independent prognostic factors negatively affecting survival.

### The influence of plasma sPD-L1 combined with STAT3 levels on DLBCL patient prognosis

**Figure 3:**
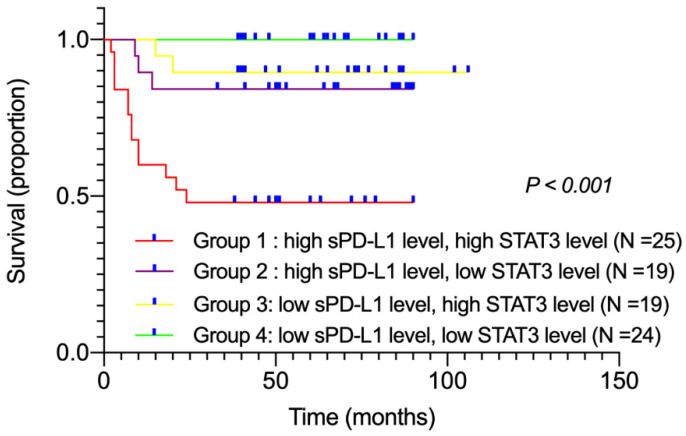
The influence of plasma sPD-L1 and STAT3 levels on DLBCL patient prognosis.

Among all patients, twenty-five displayed high sPD-L1 and high STAT3 levels (group 1), nineteen showed high sPD-L1 and low STAT3 levels (group 2), nineteen exhibited low sPD-L1 and high STAT3 levels (group 3), and twenty-four showed low sPD-L1 and low STAT3 levels (group 4). We performed survival analyses to compare these four groups according to their combination of sPD-L1 and STAT3 levels. Kaplan-Meier survival curves showed that group 1 patients had the worst OS compared with other groups (*χ^2^* = 26.289, *P* < 0.001; Figure 3). Moreover, subgroup analysis was performed. Comparing groups 3 and 4, we found that in patients with low sPD-L1 level, the survival had no significant difference with either high or low STAT3 level (*P* = 0.107). However, on comparing groups 1 and 2, we found that in patients with high sPD-L1 level, those with high STAT3 level had poorer survival rates than patients with low STAT3 level (*P* = 0.013). These findings suggested that if high STAT3 level led to the upregulation of sPD-L1 level, patients had the worst prognosis. If high STAT3 level did not lead to the upregulation of sPD-L1 level, there was no significant difference in patient survival (*P* = 0.107). According to present reports, overexpression of STAT3 may upregulate the expression of PD-L1 and relate to poor overall survival 26, 27. Our data also revealed that simultaneously high levels of plasma sPD-L1 and STAT3 were associated with poor prognosis in DLBCL patients.

### Association between plasma sPD-L1 and PD-L1 expression in tumor tissue

**Figure 4:**
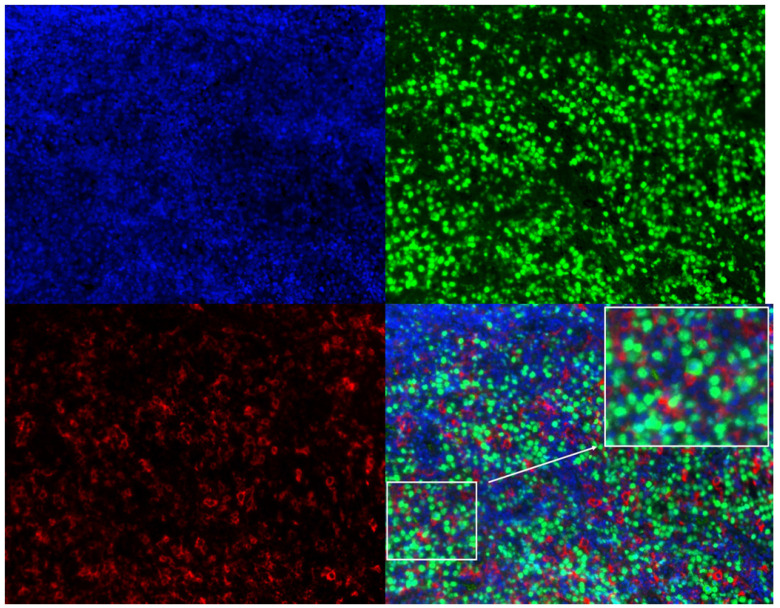
Three-color multispectral and separated individual spectral images of multiplexed immunofluorescence staining with DAPI (blue), PAX-5 (green), and PD-L1 (red). 200× magnification.

**Figure 5:**
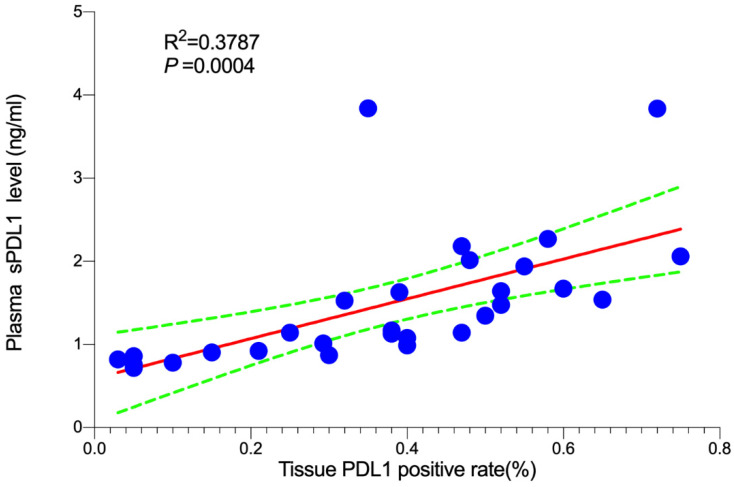
Association between plasma sPD-L1 and PD-L1 expression in tumor tissue.

In order to determine the correlation between plasma sPD-L1 level and PD-L1 expression in tumor tissues, we conducted a correlation analysis. Twenty-nine matched FFPE specimens from all patients were available prior to systemic therapy. Three-color multispectral and separated individual spectral images within the same FFPE tumor section are shown in Figure 4. The linear regression analysis showed that plasma sPD-L1 levels positively correlated with tissue PD-L1 expression (R^2^ = 0.3787, *P* = 0.0004, Figure 5).

## Discussion

A series of studies have recently reported that elevated sPD-L1 or STAT3 is associated with a poorer prognosis 17-18, 28-32, 34. Furthermore, it has been reported that multiple oncogenic pathways lead to expression of PD-L1 by upregulating STAT3 expression 25-26. However, the effect of plasma sPD-L1 combined with STAT3 on the prognosis in DLBCL patients remains unknown.

In our study, we showed that high sPD-L1 levels [HR (95% CI) = 6.284 (1.390-28.397), *P* = 0.017)] and high STAT3 levels [(HR (95% CI) = 4.158 (1.182-14.627), *P* = 0.026)] were the independent prognostic factors negatively affecting survival. We also found that the PFS and OS for high sPD-L1 level group was poorer than that for low sPD-L1 level group in all patients as well as in the non-GCB and GCB-DLBCL subgroups. As demonstrated previously 29, PFS and OS for high STAT3 level group was poorer than that for low STAT3 level group in all patients as well as in the non-GCB DLBCL, but not in the GCB-DLBCL subgroups. In addition, patients with advanced stage, IPI grades > 2, non-GCB subtype, elevated LDH and β2-M levels had higher sPD-L1 level, which also suggests that sPD-L1 levels have a potential of predicting DLBCL progression. Elevated STAT3 level was related to the advanced stage (*P* = 0.041) and IPI score (*P* = 0.044). These findings were consistent with previous reports 17-18, 29. It has been widely confirmed that DEL exhibits poor prognosis. Therefore, we investigated the correlation between DEL and the level of plasma sPD-L1 and/or STAT3 and found that patients with DEL had higher sPD-L1 levels, but no difference was observed for plasma STAT3 levels. In addition, we did not observe a relationship between plasma sPD-L1 and STAT3 in this study. The reason may be that the sPD-L1 levels were regulated by not only STAT3, but also many other different pathways. Importantly, we found that patients with DLBCL having high levels of sPD-L1 and STAT3 had the worst OS (*P* < 0.001) compared with all other patients. Interestingly, we also found that there was no significant difference in OS in patients with DLBCL having low sPD-L1 levels and high or low STAT3 levels (*P* = 0.107). However, the patients with high sPD-L1 and STAT3 levels had poorer OS compared with those having high sPD-L1 levels and low STAT3 levels (*P* = 0.013). These findings also suggest that we can stratify DLBCL patients according to plasma sPD-L1 and STAT3 levels into different risk groups.

The correlation between PD-L1 expression in the tumor tissue and plasma sPD-L1 is controversial 17, 19-20. In order to investigate the association between sPD-L1 level and tissue PD-L1 expression in DLBCL, the expression of tissue PD-L1 in few matched patients was measured. We found that plasma sPD-L1 levels positively correlated with tissue PD-L1 expression (*R^2^* = 0.3787, *P* = 0.0004, Figure 5). However, it has been reported that there is no correlation between sPD-L1 level and PD-L1 expression in tumor tissue of patients with DLBCL 17. In addition to the heterogeneity between tumors, another possibility for these controversial observations may also be attributed to different sources of PD-L1 antibodies. Therefore, these findings suggest that plasma sPD-L1 rather than tissue PD-L1 levels have a potential to predict prognosis for some subtypes of tumors. However, the small sample size of this study may influence the reliability of the conclusions; thus, a larger cohort of DLBCL patients is needed for further validation.

## Conclusions

In summary, the levels of plasma sPD-L1 and plasma STAT3 were independent prognostic factors negatively affecting survival, and high levels of both showed a significantly worse survival rate compared with that by any one high or low level of both. In the era of personalized diagnosis and treatment, combined measurement of the levels of plasma sPD-L1 and STAT3 may be a promising prognostic strategy for DLBCL patients.

## Figures and Tables

**Table 1:** Association of plasma sPD-L1 and STAT3 levels with the clinical characteristics of patients with DLBCL

Clinical Parameters	n	sPD-L1 level				STAT3 level			
		High (%)	Low (%)	χ^2^	*P* value	High (%)	Low (%)	χ^2^	*P* value
Total	87	44 (50.6)	43 (49.4)			44 (50.6)	43 (49.4)		
**Gender**									
Male	41	20 (48.8)	21 (51.2)	0.1	0.752	19 (46.3)	22 (53.7)	0.556	0.456
Female	46	24 (52.2)	22 (47.8)			25 (54.3)	21 (45.7)		
**Age (years)**									
≤ 60	59	30 (50.8)	29 (49.2)	0.005	0.941	29 (49.2)	30 (50.8)	0.148	0.700
> 60	28	14 (50)	14 (50)			15 (53.6)	13 (46.4)		
**Subtype**									
GCB	39	15 (38.5)	24 (61.5)	4.149	0.042*	22 (56.4)	17 (43.6)	0.963	0.326
Non-GCB	48	29 (60.4)	19 (39.6)			22 (45.8)	26 (54.2)		
**Clinical stage**									
I-II	45	17 (37.8)	28 (62.2)	6.107	0.013*	18 (40)	27 (60)	4.170	0.041*
III-IV	42	27 (64.3)	15 (35.7)			26 (61.9)	16 (38.1)		
**IPI grades**									
0-2	60	24 (40)	36 (60)	8.649	0.003*	26 (43.3)	34 (56.7)	4.056	0.044*
3-5	27	20 (74.1)	7 (25.9)			18 (66.7)	9 (33.3)		
**LDH level**									
Normal	49	19 (38.8)	30 (61.2)	6.248	0.012*	22 (44.9)	27 (55.1)	1.446	0.229
Elevated	38	25 (65.8)	13 (34.2)			22 (57.9)	16 (42.1)		
**β2-M level**									
Normal	70	29 (41.4)	41 (58.6)	11.988	0.001*	32 (45.7)	38 (54.3)	3.386	0.066
Elevated	17	15 (88.2)	2 (11.8)			12 (70.6)	5 (29.4)		
**Symptom**									
A	72	32 (44.4)	40 (55.6)	6.278	0.012*	35 (48.6)	37 (51.4)	0.644	0.422
B	15	12 (80)	3 (20)			9 (60)	6 (40)		
**Extra nodal involvement**									
No	31	12 (38.7)	19 (61.3)	2.712	0.1	17 (54.8)	14 (45.2)	0.350	0.554
Yes	56	32 (57.1)	24 (42.9)			27 (48.2)	29 (51.8)		
**Double-expressor lymphoma**									
Yes	28	18 (64.3)	10 (35.7)	7.701	0.021*	15 (53.6)	13 (46.3)	0.293	0.864
No	32	10 (31.3)	22 (68.7)			15 (46.9)	17 (53.1)		
NA	27	16 (59.3)	11 (40.7)			14 (51.9)	13 (48.1)		
**Clinical response**									
CR/PR	50	18 (36)	32 (64)	9.991	0.002*	23 (46)	27 (54)	0.984	0.321
SD/PD	37	26 (70.3)	11 (29.7)			21 (56.8)	16 (43.2)		

sPD-L1; soluble programmed cell death ligand 1; STAT3, signal transducer and activator of transcription 3; DLBCL, diffuse large B cell lymphoma; GCB, germinal center B-cell; IPI, international prognostic index; LDH, lactate dehydrogenase; β2-M, beta 2 microglobulin, CR/PR, complete response/partial response; SD/PD, stable disease/progressed disease. *P < 0.05.

**Table 2:** Univariate and multivariate analysis of prognostic factors in patients with DLBCL

Clinical Parameters	Univariate analysis		Multivariate analysis	
	χ^2^	*P* value	HR (95% CI)	*P* value
Gender (Male v Female)	1.922	0.166		
Age, years (≤ 60 v > 60)	0.550	0.458		
Clinical stage (I-II v III-IV)	3.483	0.062		
LDH level (Normal v Elevated)	5.515	0.019*		
β2-M level (Normal v Elevated)	11.232	0.001*		
IPI grades (0-2 v 3-5)	15.672	0.000**	3.121 (1.124-8.669)	0.029*
Extra nodal involvement (No v Yes)	0.027	0.869		
Symptom (A v B)	8.660	0.003*		
Subtype (GCB v non-GCB)	0.003	0.959		
DEL (Yes v No)	4.439	0.035*		
sPD-L1 level (Low v High)	13.708	0.000**	6.284 (1.390-28.397)	0.017*
STAT3 level (Low v High)	9.780	0.002*	4.158 (1.182-14.627)	0.026*

CI, confidence interval; HR, hazard ratio; sPD-L1; soluble programmed cell death ligand 1; STAT3, signal transducer and activator of transcription 3; DLBCL, diffuse large B cell lymphoma; LDH, lactate dehydrogenase; β2-M, beta 2 microglobulin; IPI, international prognostic index; DEL, double-expressor lymphoma. *P < 0.05, **P < 0.001.

## Abbreviations

PD-1/PD-L1: programmed cell death 1/programmed cell death ligand 1
p-STAT3: phosphorylated signal transducer and activator of transcription 3
DLBCL: diffuse large B cell lymphoma
NHL: non-Hodgkin's lymphoma
ELISA: enzyme linked immunosorbent assay
FFPE: formalin-fixed paraffin-embedded
PFS: progression-free survival
OS: overall survival
GCB: germinal center B-cell
non-GCB: non-germinal center B-cell
IPI: international prognostic index
DEL: double-expressor lymphoma
DHL: double hit lymphoma
THL: triple hit lymphoma

## References

[1] Cultrera JL, Dalia SM (2012). Diffuse large B-cell lymphoma: current strategies and future directions. Cancer Control.

[2] Swerdlow SH, Campo E, Pileri SA, Harris NL, Stein H, Siebert R (2016). The 2016 revision of the World Health Organization classification of lymphoid neoplasms. Blood.

[3] Song Y, Gao Q, Zhang H, Fan L, Zhou J, Zou D (2020). Treatment of relapsed or refractory classical Hodgkin lymphoma with the anti-PD-1, tislelizumab: results of a phase 2, single-arm, multicenter study. Leukemia.

[4] Topalian SL, Hodi FS, Brahmer JR, Gettinger SN, Smith DC, McDermott DF (2012). Safety, activity, and immune correlates of anti-PD-1 antibody in cancer. The New England Journal of Medicine.

[5] Forde PM, Chaft JE, Smith KN, Anagnostou V, Cottrell TR, Hellmann MD (2018). Neoadjuvant PD-1 blockade in resectable lung cancer. The New England Journal of Medicine.

[6] Wolchok JD, Chiarion-Sileni V, Gonzalez R, Rutkowski P, Grob J-J, Cowey CL (2017). Overall survival with combined nivolumab and ipilimumab in advanced melanoma. The New England Journal of Medicine.

[7] Topalian SL, Sznol M, McDermott DF, Kluger HM, Carvajal RD, Sharfman WH (2014). Survival, durable tumor remission, and long-term safety in patients with advanced melanoma receiving nivolumab. Journal of Clinical Oncology.

[8] Keir ME, Butte MJ, Freeman GJ, Sharpe AH (2008). PD-1 and its ligands in tolerance and immunity. Annual Review of Immunology.

[9] Kim S, Kim M-Y, Koh J, Go H, Lee DS, Jeon YK (2015). Programmed death-1 ligand 1 and 2 are highly expressed in pleomorphic carcinomas of the lung: comparison of sarcomatous and carcinomatous areas. European Journal of Cancer.

[10] Cedrés S, Ponce-Aix S, Zugazagoitia J, Sansano I, Enguita A, Navarro-Mendivil A (2015). Analysis of expression of programmed cell death 1 ligand 1 (PD-L1) in malignant pleural mesothelioma (MPM). PLoS ONE.

[11] Chen BJ, Chapuy B, Ouyang J, Sun HH, Roemer MGM, Xu ML (2013). PD-L1 expression is characteristic of a subset of aggressive B-cell lymphomas and virus-associated malignancies. Clinical Cancer Research.

[12] Kwon D, Kim S, Kim P-J, Go H, Nam SJ, Paik JH (2016). Clinicopathological analysis of programmed cell death 1 and programmed cell death ligand 1 expression in the tumour microenvironments of diffuse large B cell lymphomas. Histopathology.

[13] Frigola X, Inman BA, Lohse CM, Krco CJ, Cheville JC, Thompson RH (2011). Identification of a soluble form of B7-H1 that retains immunosuppressive activity and is associated with aggressive renal cell carcinoma. Clinical Cancer Research.

[14] Patel SP, Kurzrock R (2015). PD-L1 expression as a predictive biomarker in cancer immunotherapy. Molecular Cancer Therapeutics.

[15] Dong L, Lv H, Li W, Song Z, Li L, Zhou S (2016). Co-expression of PD-L1 and p-AKT is associated with poor prognosis in diffuse large B-cell lymphoma via PD-1/PD-L1 axis activating intracellular AKT/mTOR pathway in tumor cells. Oncotarget.

[16] Yang J, Hu G (2019). Significance of PD-L1 in the diagnosis and treatment of B-cell malignant lymphoma. Oncology Letters.

[17] Rossille D, Gressier M, Damotte D, Maucort-Boulch D, Pangault C, Semana G (2014). High level of soluble programmed cell death ligand 1 in blood impacts overall survival in aggressive diffuse large B-cell lymphoma: results from a French multicenter clinical trial. Leukemia.

[18] Wang L, Wang H, Chen H, Wang W-d, Chen X-Q, Geng Q-R (2015). Serum levels of soluble programmed death ligand 1 predict treatment response and progression free survival in multiple myeloma. Oncotarget.

[19] Nagato T, Ohkuri T, Ohara K, Hirata Y, Kishibe K, Komabayashi Y (2017). Programmed death-ligand 1 and its soluble form are highly expressed in nasal natural killer/T-cell lymphoma: a potential rationale for immunotherapy. Cancer Immunology, Immunotherapy.

[20] Zhang X, Liu L, Zhou S, Zhao K, Song Z, Hu G (2019). Plasma soluble programmed death ligand 1 levels predict clinical response in peripheral T-cell lymphomas. Hematological Oncology.

[21] Phelan JD, Young RM, Webster DE, Roulland S, Wright GW, Kasbekar M (2018). A multiprotein supercomplex controlling oncogenic signalling in lymphoma. Nature.

[22] Rosenquist R, Beà S, Du M-Q, Nadel B, Pan-Hammarström Q (2017). Genetic landscape and deregulated pathways in B-cell lymphoid malignancies. Journal of Internal Medicine.

[23] Xu Z-Z, Xia Z-G, Wang A-H, Wang W-F, Liu Z-Y, Chen L-Y (2013). Activation of the PI3K/AKT/mTOR pathway in diffuse large B cell lymphoma: clinical significance and inhibitory effect of rituximab. Annals of Hematology.

[24] Horlad H, Ma C, Yano H, Pan C, Ohnishi K, Fujiwara Y (2016). An IL-27/Stat3 axis induces expression of programmed cell death 1 ligands (PD-L1/2) on infiltrating macrophages in lymphoma. Cancer Science.

[25] Song TL, Nairismägi M-L, Laurensia Y, Lim J-Q, Tan J, Li Z-M (2018). Oncogenic activation of the STAT3 pathway drives PD-L1 expression in natural killer/T-cell lymphoma. Blood.

[26] Marzec M, Zhang Q, Goradia A, Raghunath PN, Liu X, Paessler M (2008). Oncogenic kinase NPM/ALK induces through STAT3 expression of immunosuppressive protein CD274 (PD-L1, B7-H1). Proceedings of the National Academy of Sciences of the USA.

[27] Yu H, Pardoll D, Jove R (2009). STATs in cancer inflammation and immunity: a leading role for STAT3. Nature Reviews Cancer.

[28] Wu P, Wu D, Zhao L, Huang L, Shen G, Huang J (2016). Prognostic role of STAT3 in solid tumors: a systematic review and meta-analysis. Oncotarget.

[29] Huang X, Meng B, Iqbal J, Ding BB, Perry AM, Cao W (2013). Activation of the STAT3 signaling pathway is associated with poor survival in diffuse large B-cell lymphoma treated with R-CHOP. Journal of Clinical Oncology.

[30] Wu ZL, Song YQ, Shi YF, Zhu J (2011). High nuclear expression of STAT3 is associated with unfavorable prognosis in diffuse large B-cell lymphoma. Journal of Hematology and Oncology.

[31] Johnson DE, O'Keefe RA, Grandis JR (2018). Targeting the IL-6/JAK/STAT3 signalling axis in cancer. Nature Reviews Clinical Oncology.

[32] Chong PSY, Chng W-J, de Mel S (2019). STAT3: a promising therapeutic target in multiple myeloma. Cancers.

[33] Ok CY, Chen J, Xu-Monette ZY, Tzankov A, Manyam GC, Li L (2014). Clinical implications of phosphorylated STAT3 expression in De Novo diffuse large B-cell lymphoma. Clinical Cancer Research.

[34] Kwon HJ, Yang JM, Lee J-O, Lee JS, Paik JH (2018). Clinicopathologic implication of PD-L1 and phosphorylated STAT3 expression in diffuse large B cell lymphoma. Journal of Translational Medicine.

[35] Wang X, Zhang T, Song Z, Li L, Zhang X, Liu J (2019). Tumor CD73/A2aR adenosine immunosuppressive axis and tumor-infiltrating lymphocytes in diffuse large B-cell lymphoma: correlations with clinicopathological characteristics and clinical outcome. International Journal of Cancer.

